# Stem stiffness functionality in a submerged canopy patch under oscillatory flow

**DOI:** 10.1038/s41598-023-28077-2

**Published:** 2023-02-02

**Authors:** Aina Barcelona, Jordi Colomer, Teresa Serra

**Affiliations:** Department of Physics, University of Girona, 17071 Girona, Spain

**Keywords:** Environmental sciences, Ocean sciences, Engineering

## Abstract

Seagrass canopies are coastal ecosystems that are able to modify the abiotic environment through their architectural structure. They have different structural parameters, such as plant stem stiffness, patch length and canopy density, all of which determine their overall functionality in modifying the seafloor hydrodynamics within coastal areas. To determine the interaction between hydrodynamics and the canopy structure, a set of laboratory experiments were carried out with both rigid and flexible stems for different canopy densities, patch lengths and wave frequencies. In the upper part of the canopy, flexible plants move with the flow without generating drag or producing turbulent kinetic energy, while rigid plants generate drag and produce turbulent kinetic energy. In the inner canopy layer, both types of plants behave like rigid stems and produce turbulent kinetic energy. A non-dimensional model based on the turbulent kinetic energy, the wave velocity and the plant characteristics is presented to describe the behaviour of flexible and rigid plants under an oscillating flow. Flexible plants behave in a stiffer manner under high wave frequencies than under low wave frequencies, thus making their behaviour closer to that of rigid plant stems. This difference between both canopy structures can explain their distribution in the environment, with rigid canopies being more extended in more sheltered regions while flexible plants are characteristic of more exposed regions with high flow energy.

## Introduction

Seagrasses are valuable coastal ecosystems that protect the seabed from waves and currents^1,2^. They also provide habitats for aquatic life, improve water quality, sequester carbon, and stabilize sediment^3–5^. However, they are situated in regions where anthropogenic activities like anchoring, dredging, trawling, or sewage outflow cause their decline^6,7^. Human pressure has produced a 30–60% decline in seagrasses^8^. In some places, seagrasses have completely disappeared, while in others seagrass landscapes have changed from large continuous meadows to fragmented canopies^9^, where a patchy distribution of plants dominates the seascape.

There is a lack of data concerning the hydrodynamics for all types of canopies, patch characteristics and the degree of landscape fragmentation^10^. While the hydrodynamics in continuous meadows is expected to be spatially homogeneous, in fragmented meadows^11^ it is likely to be spatially heterogeneous. In addition, the increase in the degree of meadow’s fragmentation also increases the overall turbulent kinetic energy, thus enhancing mixing for a greater sediment resuspension^11^. Therefore, it is expected that canopy fragmentation increases meadow vulnerability under external pressures^12^.

Considering that fragmented landscape seagrasses are made up of patches of different sizes^13^, patch length, then, is expected to determine the hydrodynamics in fragmented meadows. Interspersed within vegetation of fragmented meadows are gaps (i.e., zones without vegetation). The larger the gap, the greater the turbulent kinetic energy and wave velocity within that gap^14^ is. However, for a certain gap size, the degree of meadow fragmentation has not been found to impact the hydrodynamics^14^. In contrast, the degree of fragmentation does impact canopy density at canopy interfaces near a gap^15^. These results reveal the need for more studies into the effect fragmentation has on the hydrodynamics within a fragmented meadow.

Vegetation produces a flow resistance that can differ depending on the plants’ distinct structural characteristics, i.e., stem diameter, height, thickness, whether plants are submerged or emergent, their flexibility and horizontal distribution (density, staggered or random). Laboratory studies using models of rigid stems under oscillatory flows have shown that the wave velocity attenuation is greater for emergent stems than submerged ones^16^. Many of the studies into the hydrodynamics in rigid meadows under oscillatory and unidirectional flows have been conducted in laboratory flumes^2,17,18^ in order to understand the role seagrasses play in sheltering the seabed. In addition, studies of the hydrodynamics in flexible meadows have also been carried out in the laboratory to better mimic seagrasses and understand the effect of flexibility. A flexible plant exhibits different configurations compared to rigid plant stems as they can remain erect, sway or be prone^19^. The turbulent kinetic energy within a meadow of submerged flexible plants has been found to depend on *A*_*w*_*/S*, i.e., the ratio between the orbital wave excursion *A*_*w*_ and the plant-to-plant distance S^20^. For *A*_*w*_*/S* > 1 the turbulent kinetic energy (*TKE*) increases with *A*_*w*_*/S* whereas it remains constant for *A*_*w*_*/S* < 1^20^. It must be pointed out that, despite the different structure of rigid and flexible stems, for low flow velocities the behaviour of flexible plant stems can be close to that of rigid plant stems due to the small amount of bending involved. However, the behaviour of patches of vegetation with different sizes of flexible and rigid plants has yet to be studied.

Understanding the relationship between all of the above-mentioned structural characteristics of the vegetation, along with the hydrodynamics might, offer clues as to what the optimal patch length scales, meadow densities or plant distributions are that could explain the resilience exhibited by some meadows. Some studies reveal that there are positive ecological interactions that favour the success of seagrass restoration^21^. The authors of these studies note that canopy density might play a positive dependence role, thus improving the survival of a seagrass population. Other structural parameters might likewise play critical roles in facilitating restoration projects, for instance, the minimum patch size that a patch of vegetation has to have or the arrangements of the stems in the patch. Hydrodynamics and plant characteristics have been found to determine sediment scouring that in turn can compromise seagrass restoration strategies^22^. High turbulent flows can lead to sediment scouring around plant stems.

Bouma et al.^23^ compared the role of *Spartina alternifora* plants to that of *Zostera noltii*. (*Spartina alternifora* shoots are much stiffer than *Zostera noltii* shoots) in terms of their capacity to dissipate hydrodynamic forces) and found that dissipation was three times higher in vegetation with stiffer leaves than in vegetation with flexible leaves. They hypothesized that the drag exerted by the flow limits how far off the coast *Spartina* can grow. In more exposed areas, where the hydrodynamics are strong, other drag-minimizing species like *Zostera noltii* will grow, generating a sharp interface or transition between the extension of both types of ecological engineers. Therefore, seagrasses need to withstand hydrodynamic forces so that the costs (through drag) and benefits (their ability to modify the habitat conditions) are advantageous for their survival^23^. In addition, seagrasses have been found to have the capacity to adapt to certain environmental conditions by acclimation of their flexibility^23^. Paul and de los Santos^24^ found that *Zostera marina* leaves were more rigid in summer than in winter and in deep sheltered zones than in shallow exposed zones where they presented more flexible leaves.

Hydrodynamics being modified by different types of plants (flexible or rigid) and patch lengths is still of concern and the role patch length plays for different plants' stiffness needs to be investigated. In the present study, the behaviour of single patches of different sizes formed by a random distribution of rigid or flexible plants under oscillatory conditions has been investigated. To this purpose, laboratory experiments were carried out in a flume using models of both rigid and flexible plants. To determine the behaviour of plants (rigid and flexible) under different hydrodynamic conditions two wave frequencies were considered. In addition, previous results obtained by other authors for a fixed patch length have been included in the study to provide a wider range of flow conditions and to compare between rigid and flexible plants. The modification of the hydrodynamics on the vertical axis by each type of plant and for each wave field was studied through the behaviour of the turbulent kinetic energy (*TKE*). The *TKE* can then be an indicator of the sediment resuspension in each set-up and provide clues on the possible resilience of seagrasses under different hydrodynamic conditions.

## Results

**Figure 1:**
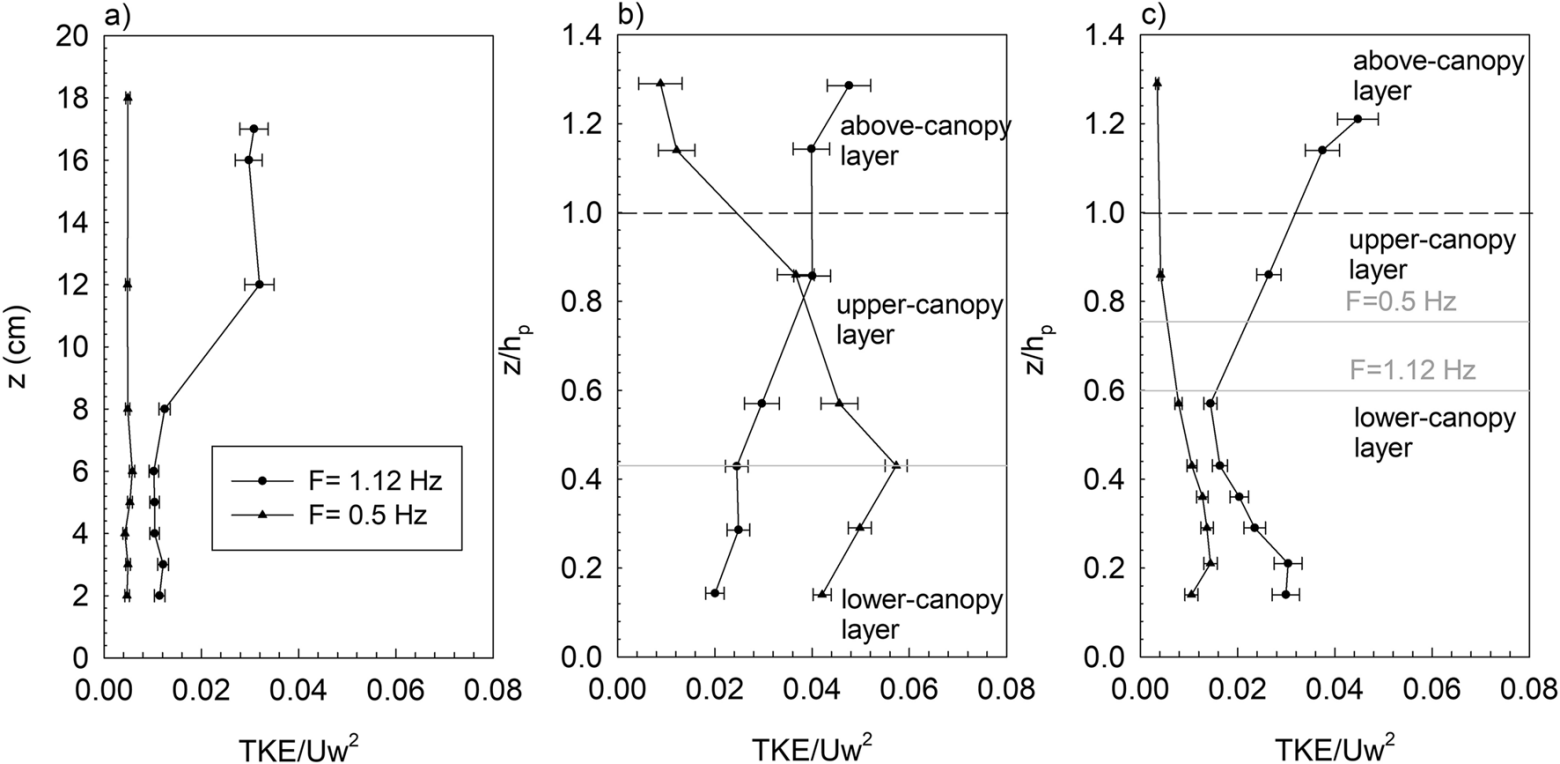
*TKE/Uw*^*2*^ vertical profiles (**a**) versus z for non-vegetated set ups, and versus *z/h*_*p*_ for (**b**) rigid vegetation and (**c**) flexible vegetation for the two wave frequencies studied *f* = 1.12 Hz (circles) and (**b**) *f* = 0.5 Hz (triangles). The horizontal dashed lines in (**b**) and (**c**) represent the height of the plant leaf for flexible plants (*h*_*p*_) or the height of the plant stem for rigid plants. The vegetated experiments presented here correspond to cases with a patch length of 238 cm and SPF = 10%*.* The horizontal grey lines represent the interface between the upper-canopy layer and the lower-canopy layer for both types of vegetation.

The vertical profiles of *TKE/U*_*w*_^*2*^ presented different patterns depending on the wave frequency (Fig. 1a). For the non-vegetated set ups, and for the wave frequency of 1.12 Hz, *TKE/U*_*w*_^*2*^ presented a constant value at the top of the water column. Below this layer, a gradual decrease of *TKE/U*_*w*_^*2*^ was noted until a constant value situated at the bottom layer was observed. In contrast, for the wave frequency of 0.5 Hz, *TKE/U*_*w*_^*2*^ presented a constant value with z (Fig. 1a). From the vertical profiles of the normalized turbulent kinetic energy (*TKE/U*_*w*_^*2*^) in the vegetated set-ups, three layers could be distinguished. The above-canopy layer (ACL) corresponded to the layer above the maximum canopy height (h_p_, determined as the leaf length for flexible plants and the stem length for the rigid canopy). In this layer *TKE/U*_*w*_^*2*^ presented three behaviours depending on the wave frequency. In the ACL, for the wave frequency of 1.12 Hz, *TKE/U*_*w*_^*2*^ tended to decrease (rigid, Fig. 1b) or remain constant (flexible, Fig. 1c) moving upwards from the canopy. In contrast, for the wave frequency of 0.5 Hz, *TKE/U*_*w*_^*2*^ increased with z/h_p_ for both rigid and flexible vegetation. From the *TKE/U*_*w*_^*2*^ profiles, a second interface could be observed. For the rigid canopy model, an interface between the upper-canopy layer (Fig. 1b), and which was situated at the same depth (*z/h*_*p*_ = 0.44) for both wave frequencies, was observed. In the lower-canopy layer (LCL), *TKE/U*_*w*_^*2*^ presented a smaller decrease with z/h_p_ in the case of the wave frequency of 1.12 Hz compared with the upper-canopy layer (UCL). In contrast, for the wave frequency of 0.5 Hz and for the LCL, *TKE/U*_*w*_^*2*^ decreased with *z/h*_*p*_ contrary to its behaviour in the UCL. For the flexible vegetation, the interface between the UCL and the LCL depended on the wave frequency (Fig. 1c), and the interface was situated at the depth of the effective plant height *h*_*v*_ (i.e., the height of the plant bent by the wave). In the LCL, for flexible vegetation and for a both wave frequencies, *TKE/U*_*w*_^*2*^ increased downwards as *z/h*_*p*_ decreased (Fig. 1c).

**Figure 2:**
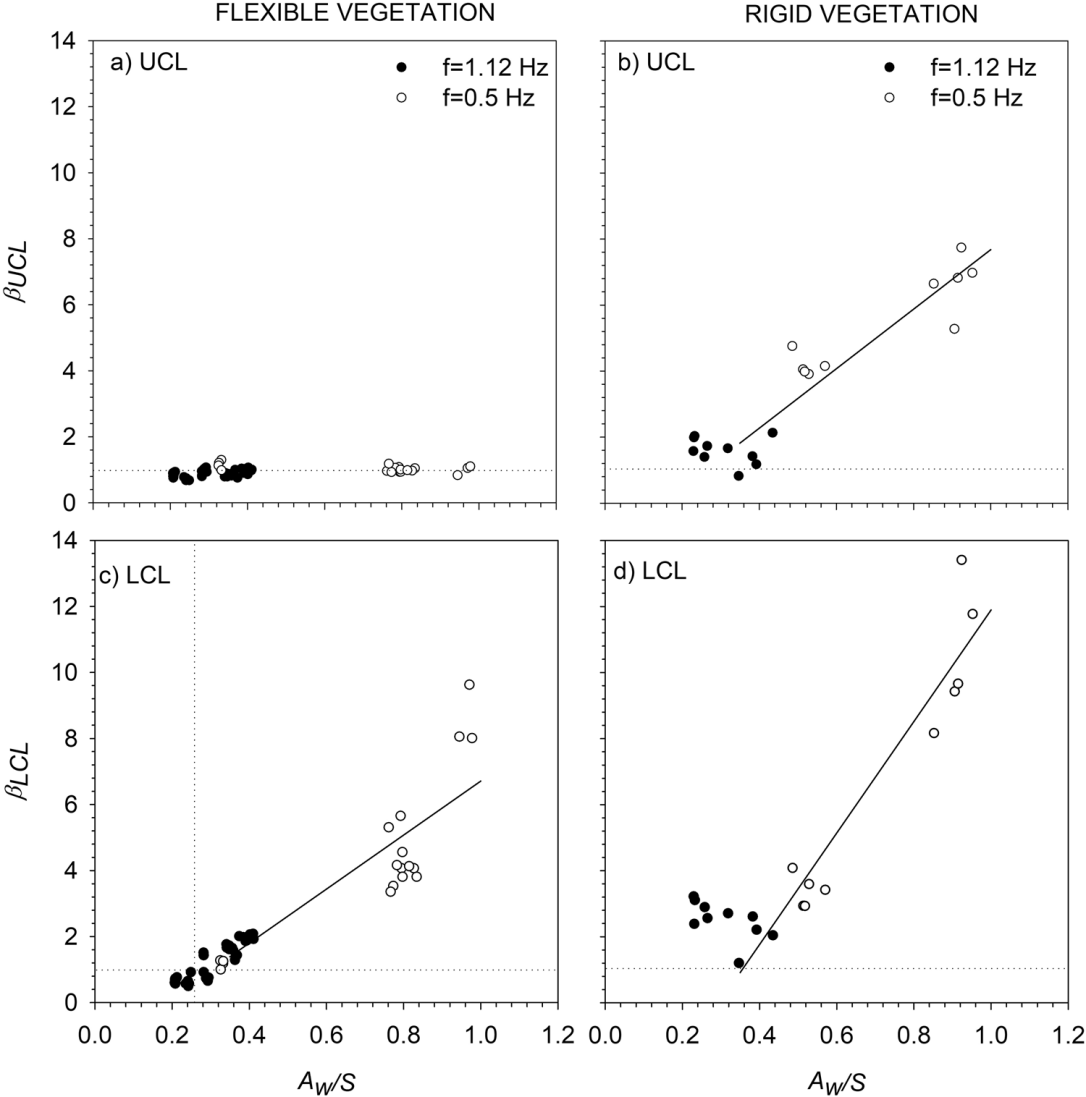
*TKE* attenuation in relation to the non-vegetated cases at the UCL (*β*_*UCL*_) for (**a**) flexible vegetation (**a**) and for (**b**) rigid vegetation (**b**). *TKE* attenuation in relation to the non-vegetated cases at the LCL (*β*_*LCL*_) for (**c**) flexible vegetation and for (**d**) rigid vegetation. Unfilled circles correspond to *f* = 0.5 Hz, and solid black circles to *f* = 1.12 Hz. Lines correspond to the linear best fit for the cases *A*_*w*_*/S* > 0.35 when *β* increased linearly with *A*_*w*_*/S*, independent of the wave frequency. In the UCL for rigid vegetation, *β* = 9.02 × *A*_*w*_*/S*-1.34 (*R*^*2*^ = 0.8625, *p-value* < 0.01). In the LCL for rigid vegetation, *β* = 16.90 × *A*_*w*_*/S*-5.00 (*R*^*2*^ = 0.9224, *p-value* < 0.01), and for the flexible vegetation *β* = 8.19 × *A*_*w*_*/S*-1.48 (*R*^*2*^ = 0.7819, *p-value* < 0.01). Vertical dashed lines represent the x-axis position where *A*_*w*_*/S* = 0.35, and horizontal dashed lines correspond to the y-axis position where *β* = 1.

The *TKE* attenuations comparing vegetated with non-vegetated cases for both the UCL and LCL(*β*_*UCL*_ and *β*_*LCL*_), were considered for all the rigid and flexible vegetation set-ups. For the flexible vegetation, *β*_*UCL*_ was found to be nearly 1 for all *A*_*w*_*/S* (where *A*_*w*_ = *U*_*w*_/2π*f*, *U*_*w*_ is the wave velocity and *S* is the plant to plant distance, see the “Methodology” section for more information) and both frequencies (Fig. 2a). However, for the rigid vegetation, *β*_*UCL*_ increased with *A*_*w*_*/S*, from the threschold of A_w_/S > 0.35 and followed a linear trend (*β*_*UCL*_ = 9.08*A*_*w*_*/S*-1.38, R^2^ = 0.837, *p* < 0.05) (Fig. 2b).

At the LCL for flexible vegetation, the same threshold at *A*_*w*_*/S* = 0.35 was found for *β*_*LCL*_ (Fig. 2c). For *A*_*w*_*/S* < 0.35 the values of *β*_*LCL*_ were close to 1, while for *A*_*w*_*/S* > 0.35, *β*_*LCL*_ was higher than 1 (Fig. 2c). In this latter case, *β*_*LCL*_ increased with *A*_*w*_*/S* following a linear trend. Otherwise, for the rigid vegetation *β*_*LCL*_ > 1 was found for all *A*_*w*_*/S*. In this case, *β*_*LCL*_ increased with *A*_*w*_*/S* following a linear trend with a greater slople than for the flexible vegetaion case (Fig. 2d).

**Figure 3:**
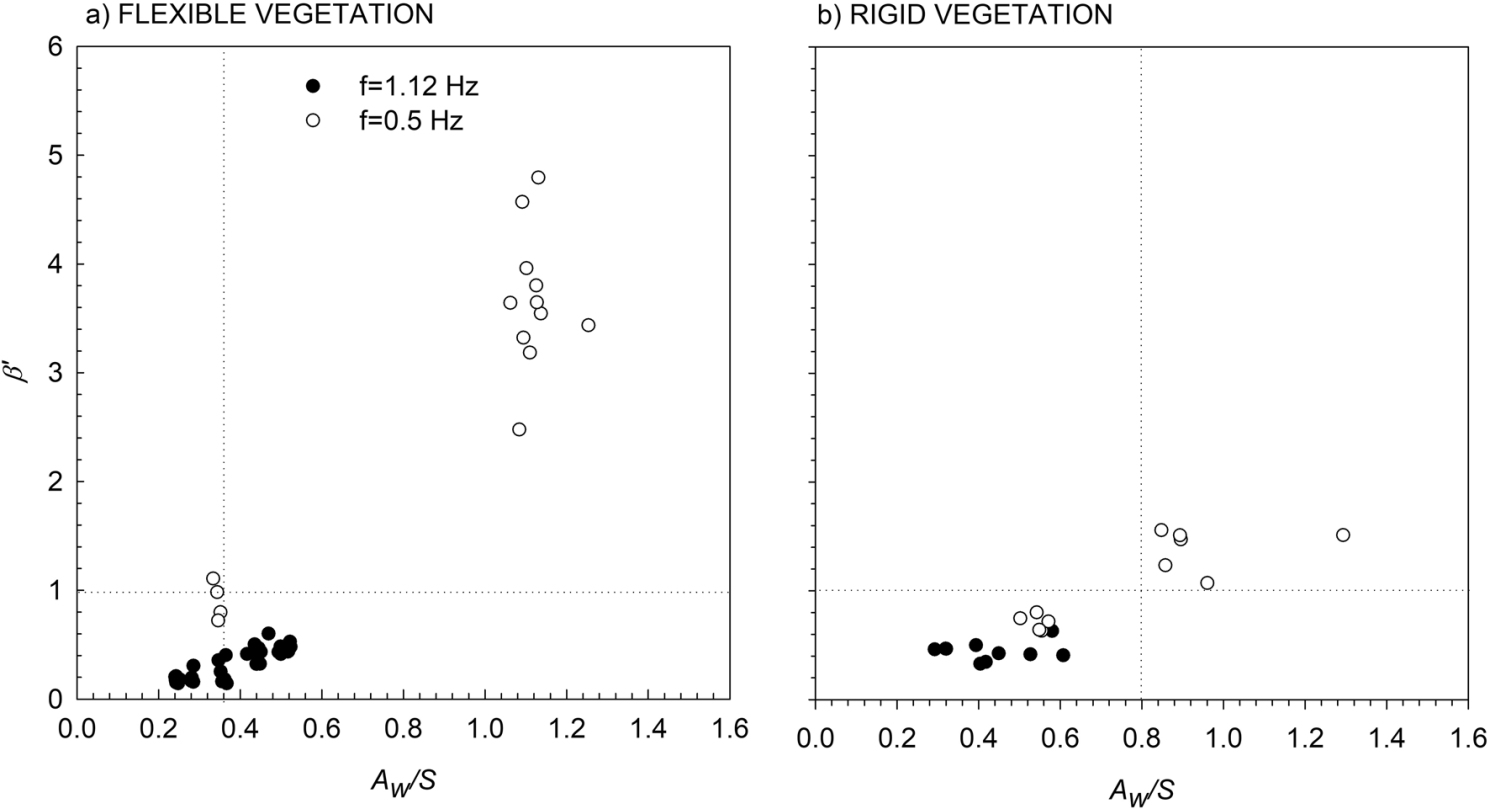
Vertical *TKE* attenuation, *β′,* for the (**a**) flexible vegetation model and (**b**) rigid vegetation model. Unfilled circles correspond to *f* = 0.5 Hz, and solid black circles correspond to *f* = 1.12 Hz. The vertical dashed lines indicate the threshold of *A*_*w*_*/S* for each type of plant, and the horizontal dashed line represents the y-axis value of *β′* = 1.

For the flexible vegetation, the vertical attenuation of the *TKE* (*β′*, see the “Methodology” for its definition) was lower than 1 for *f* = 1.12 Hz, while for *f* = 0.5 Hz two different behaviours were found: for *A*_*w*_*/S* < 0.35 ***β****′* ≈ 1 whereas for *A*_*w*_*/S* > 0.35 ***β****′* > 1 (Fig. 3a). For the rigid vegetation two behaviours were also found: for *A*_*w*_*/S* < 0.8 ***β****′* < 1, which included all the cases with *f* = 1.12 Hz and some cases of *f* = 0.5 Hz; meanwhile for *A*_*w*_*/S* > 0.8 ***β****′* > 1, which included the rest of the cases of *f* = 0.5 Hz (Fig. 3b).

**Figure 4:**
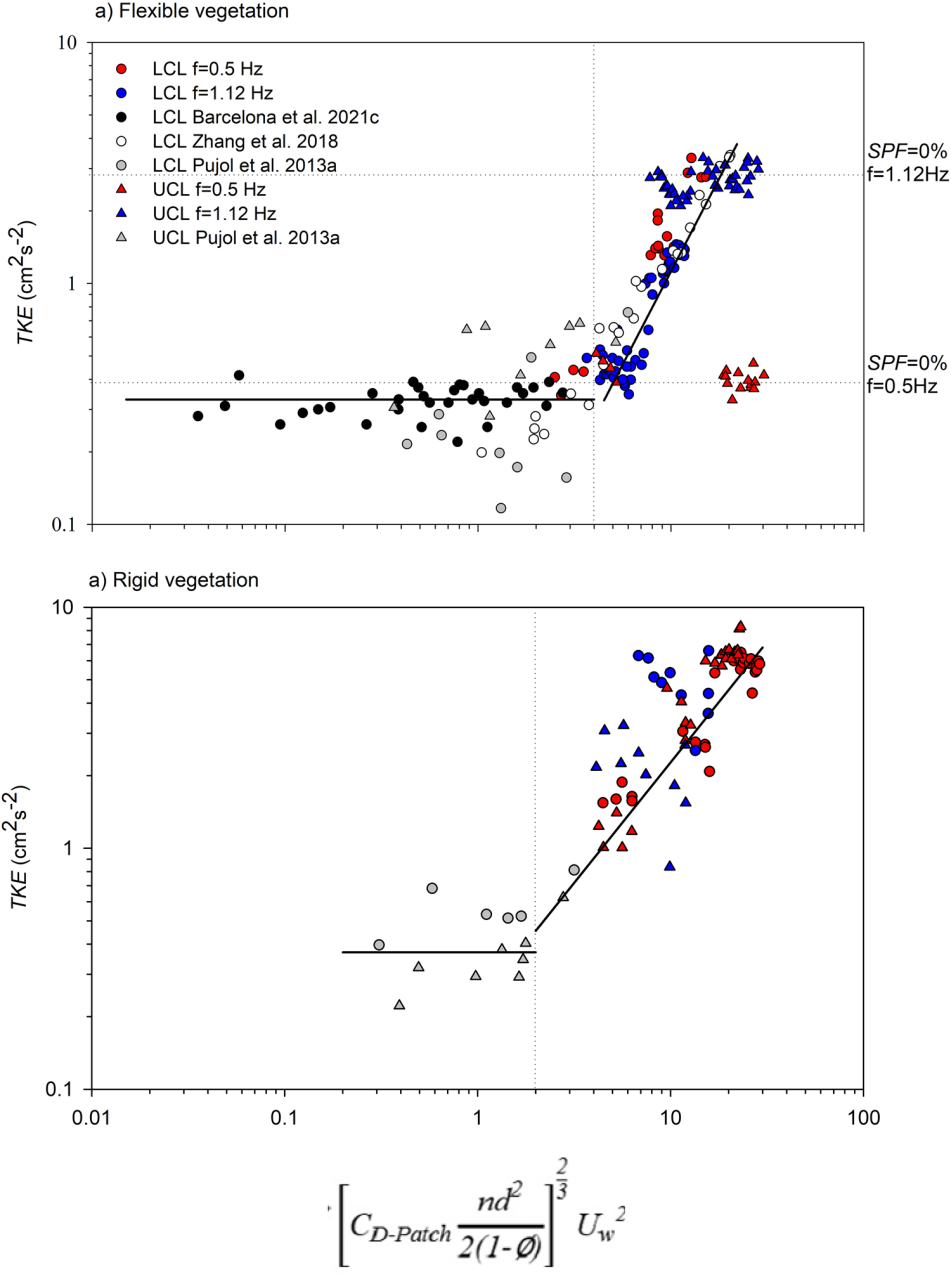
*TKE* versus ${\left[{C}_{D-Patch}\frac{n{d}^{2}}{2(1-\varnothing )}\right]}^\frac{2}{3}{{U}_{w}}^{2}$ for (**a**) flexible and (**b**) rigid vegetation. Data from Barcelona et al.^25^, and Zhang et al.^20^ for flexible vegetation have been included and data from Pujol et al.^16^ for flexible vegetation and rigid vegetation have been included as well. The vertical dashed line indicates the threshold that separated the two behaviours. The solid line corresponds to the best fit line of the data points to the model for ${\left[{C}_{D-Patch}\frac{n{d}^{2}}{2(1-\varnothing )}\right]}^\frac{2}{3}{{U}_{w}}^{2}>2$ or ${\left[{C}_{D-Patch}\frac{n{d}^{2}}{2(1-\varnothing )}\right]}^\frac{2}{3}{{U}_{w}}^{2}>4$, for both flexible and rigid plants. Horizontal dashed lines in (**a**) correspond to the *TKE* for cases without plants and for both wave frequencies.

**Figure 5:**
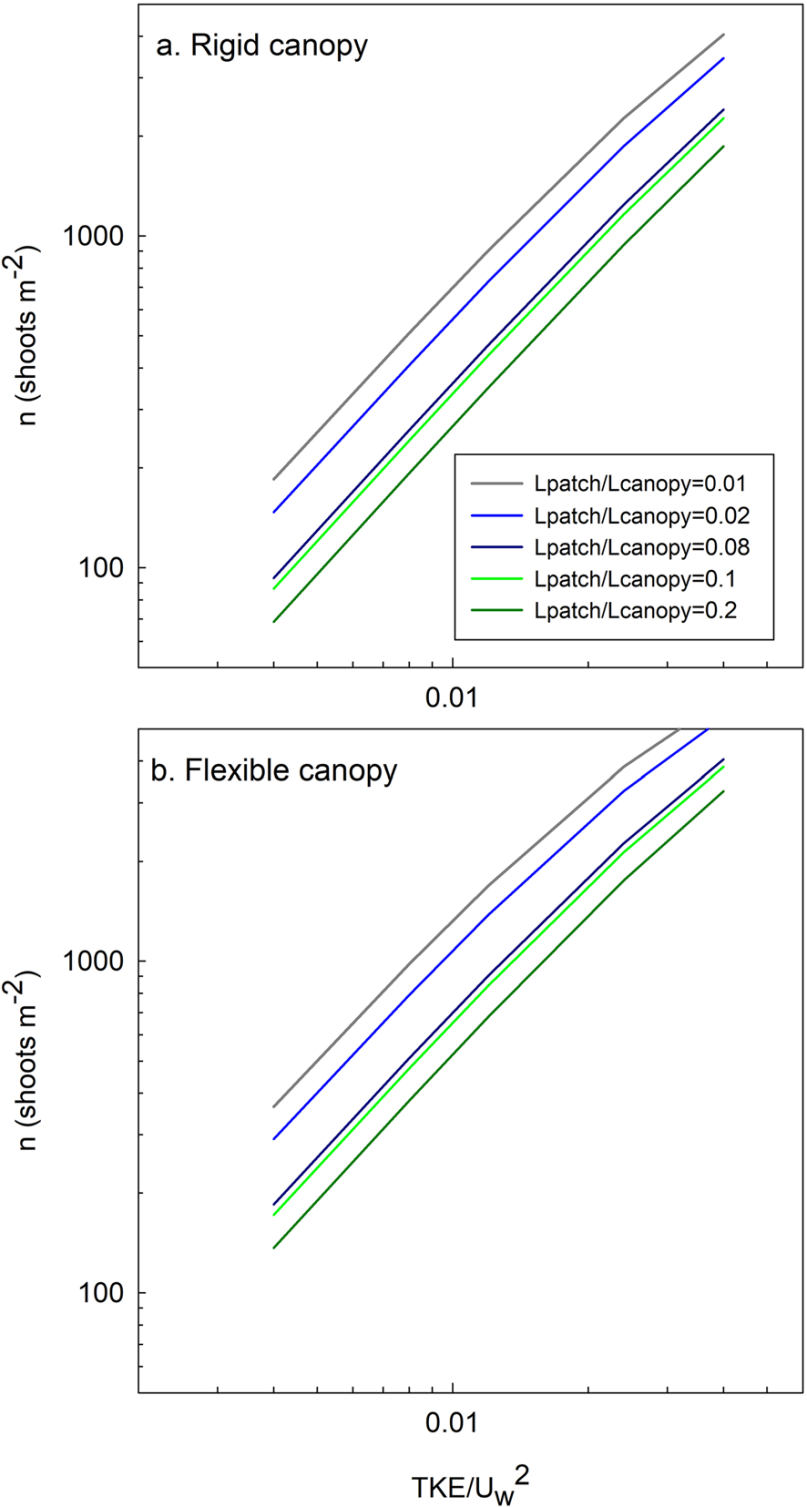
Number of shoots per m^2^ (*n*) required to begin producing *TKE* versus *TKE/U*_*w*_^*2*^ for different patch lengths and for (**a**) rigid and (**b**) flexible plant structures.

The model from Eq. (12), was used to represent the *TKE* versus ${\left[{C}_{D-Patch}\frac{n{d}^{2}}{2(1-\varnothing )}\right]}^\frac{2}{3}{{U}_{w}}^{2}$ (where *n* is the canopy density, *d* the stem diameter and the solid plant fraction is $\phi =n\frac{\pi }{4}{d}^{2}$) for all experiments carried out with both the rigid and flexible models, where ${C}_{D-Patch}= {C}_{D}{\left(\frac{{L}_{Patch}}{{L}_{Canopy}}\right)}^\frac{1}{3}$, (see the “Methodology” section for a complete description of the model). For both the flexible and rigid vegetation models, two regions could be differentiated (Fig. 4a,b). For the flexible vegetation model (Fig. 4a), and for those cases with ${\left[{C}_{D-Patch}\frac{n{d}^{2}}{2(1-\varnothing )}\right]}^\frac{2}{3}{{U}_{w}}^{2}<4$, *TKE* was constant, at *TKE* = 0.33 cm^2^ s^−2^. In contrast, for ${\left[{C}_{D-Patch}\frac{n{d}^{2}}{2(1-\varnothing )}\right]}^\frac{2}{3}{{U}_{w}}^{2}>4$ two different behaviours were found. For the UCL, *TKE* for flexible vegetation was constant, with *TKE* = 0.41 cm^2^ s^−2^ for *f* = 0.5 Hz and *TKE* = 3.10 cm^2^ s^−2^ for *f* = 1.12 Hz, corresponding to the *TKE* measured without plants (SPF = 0%) for each frequency. For the LCL, the *TKE* increased linearly (*TKE* = 0.$20{\left[{C}_{D-Patch}\frac{n{d}^{2}}{2(1-\varnothing )}\right]}^\frac{2}{3}{{U}_{w}}^{2}-0.6$, R^2^ = 0.832, *p* < 0.05) (Fig. 4a). For the rigid vegetation model (Fig. 5b), the threshold where *TKE* changed from being constant to increasing linearly from ${\left[{C}_{D-Patch}\frac{n{d}^{2}}{2(1-\varnothing )}\right]}^\frac{2}{3}{{U}_{w}}^{2}=2$. Therefore, for ${\left[{C}_{D-Patch}\frac{n{d}^{2}}{2(1-\varnothing )}\right]}^\frac{2}{3}{{U}_{w}}^{2}>2$, *TKE* followed a linear trend (*TKE* = 0.$27{\left[{C}_{D-Patch}\frac{n{d}^{2}}{2(1-\varnothing )}\right]}^\frac{2}{3}{{U}_{w}}^{2}-0.5$, R^2^ = 0.512, *p* < 0.05), while for ${\left[{C}_{D-Patch}\frac{n{d}^{2}}{2(1-\varnothing )}\right]}^\frac{2}{3}{{U}_{w}}^{2}<2$ , *TKE* remained constant with *TKE* = 0.37(Fig. 4b).

## Discussion

In coastal zones, the structural characteristics of aquatic vegetation: stiffness, canopy density, height and patch length, play a crucial role in determining their functionality as ecological engineers. Rigid canopy patches provide greater drag than flexible canopy patches do under the same hydrodynamic conditions. This result might pose some limitations for rigid canopies if they are to sustain high energy flows.

Over bare soil, (i.e., without the presence of plants), the *TKE* declines with depth for all the wave frequencies studied. These results are in accordance with previous findings by Pujol et al.^16^ and Zhang et al.^20^ who found that *TKE* decreases with depth in non-vegetated beds. However, depending on the interaction between waves and plant stems, plants can reduce the *TKE* or in contrast, they can increase it due to the drag exerted by plant stems. In this case, the flexibility of the plant also determines the attenuation of the *TKE*. Rigid plants can produce drag along the entire plant stem, whereas flexible plants behave like a blade at the top, i.e. they move back and forth with the flow, thus reducing the relative motion between the flow and the blade^26^. However, they behave like a stem at the bottom, i.e., remain stiff with an increase in their relative motion.

In this study, the vertical attenuation of the *TKE* was studied by using two attenuation parameters: vertical attenuation (*β′*) and attenuation by comparing the *TKE* with plants to the *TKE* without plants (*β*). For the rigid vegetation, the vertical attenuation *β′* is always below 1, indicating that the *TKE* in the LCL is lower than that at the UCL due to the drag produced by rigid stems in these two layers. However, for the flexible vegetation, *β′* is lower than 1 for those cases with *A*_*w*_*/S* < 0.35, accounting for all the experiments carried out for *f* = 1.12 Hz and some at 0.5 Hz. In contrast, *β′* > 1 for all the experiments with *A*_*w*_*/S* > 0.35, corresponding to some experiments carried out at *f* = 0.5 Hz. This result can be attributed to the fact that at high frequencies when *A*_*w*_*/S* > 0.35, waves interact with the canopy of flexible plants producing *TKE* along the entire plant blade (due to the wakes generated) and so the plants remain stiff (i.e., behaving more like rigid plant stems). In contrast, low wave frequencies with *A*_*w*_*/S* < 0.35 do not interact with the canopy, presenting a greater oscillatory excursion length at the top of the plant that at the bottom of it without producing wakes around the blades. In this case, flexible plants bend with the flow following a back and forth movement. These results align with the findings by van Veelen et al.^27^ who studied wave damping by vegetation with differing flexibilities. In their study they found that flexible plants swayed with the flow and did not dampen wave velocities. In contrast, rigid plants produced a greater resistance, thus damping wave velocities. Wave damping is expected to be related to the production of *TKE*, thus coinciding with the results of the current study.

The attenuation of the *TKE* in both the UCL and LCL when compared to the without-plants experiments indicated that for the experiments carried out with rigid plants, and for all the wave frequencies studied *β*_*UCL*_ > 1 and *β*_*UCL*_ > 1 for both the UCL and LCL layers, also indicating that rigid plants produced *TKE* due to the greater relative motion between the waves and the rigid stems. These results align with the conclusions of Pujol et al.^2^, who described the production of *TKE* by rigid canopies in the UCL due to the generation of stem-wake turbulence associated to a large reduction in wave velocity. For the case of a canopy of flexible plants, *β*_*UCL*_ was nearly 1 for the UCL since, at this depth, there is no plant-generated *TKE* because flexible plants swing with the flow and do not add any additional drag resistance to the movement; this behaviour could be described as a blade-like behaviour^20^. In this case, flexible plants reduce the drag to withstand the energy of the flow. This aligns with Paul and de los Santos^24^ who found that the more rigid *Zostera marina* plants acclimatise in shallower regions far from energetic flow conditions while the more flexible *Zostera marina* plants extend far out from the coast.

This behaviour observed in the UCL changed in the LCL. For the case of a canopy of flexible plants and in the LCL, *β*_*LCL*_ > 1 for cases when *A*_*w*_*/S* > 0.35, whereas *β*_*LCL*_ < 1 for cases when *A*_*w*_*/S* < 0.35. This threshold obtained for *A*_*w*_*/S* = 0.35 is equal to *A*_*w*_*/S*_*b*_ = 1 (where *S*_*b*_ is the spacing considering that stems have eight blades, (i.e., *S*_*b*_ = 1/(8* N*)^1/2^ and *N* is the stem density). This transition was also found by Zhang et al.^20^ for the inner canopy layer of flexible plants. The experiments carried out by Pujol et al.^16^ for flexible plants all corresponded to the cases *A*_*w*_*/S* < 0.35. In such conditions, single stems do not contribute to *TKE* generation, instead, stems dampen the near-bed generated *TKE* relative to the non-vegetated cases. In contrast, for flexible meadows with *A*_*w*_*/S* > 0.35, the *TKE* will be enhanced within the vegetated region relative to the non-vegetated cases. This *TKE* production determines that flexible vegetation in the LCL for *A*_*w*_*/S* > 0.35 presents stem-like behaviour similar to rigid stems. A decrease in the *TKE* within a meadow of *Posidonia oceanica* was also found by Serra et al.^14^ when compared to nearby gaps (areas without vegetation). In such cases, the canopy density was *N* = 400 stems m^−2^, *T* = 3.64 s and *U*_*w*_ = 0.01 m s^−1^, resulting in *A*_*w*_*/S* = 0.12 < 1. Granata et al.^28^ also found a vertical attenuation of *TKE* in a meadow of *Posidonia oceanica*. They compared the *TKE* above the canopy with the *TKE* within the canopy. In this case, the meadow sheltered the bed, i.e., stabilizing the sediment. Barcelona et al.^25^ studied the capture of sediment particles via a model canopy of flexible plants in a flume and found that a meadow of flexible plants enhances sedimentation compared to non-vegetated conditions.

The present study demonstrates that *TKE* production by vegetation depends on wave velocity, canopy density, the plant flexibility and patch length for both rigid and flexible vegetation models. The thresholds ${\left[{C}_{D-Patch}\frac{n{d}^{2}}{2(1-\varnothing )}\right]}^\frac{2}{3}{{U}_{w}}^{2}>4$ (undefined for flexible plants^29^) and ${\left[{C}_{D-Patch}\frac{n{d}^{2}}{2(1-\varnothing )}\right]}^\frac{2}{3}{{U}_{w}}^{2}>2$ (for rigid plants, observed in the current study) is required for the canopy to produce *TKE*. It is important to notice that the production of *TKE* holds at a lower threshold for rigid than for flexible plants, because flexible plants move with the flow. Van Veelen et al.^27^ also found that for low submergence ratios of the vegetation, like that in the current study (*h*_*p*_*/H* = 0.47), the drag produced by the canopy varies depending on the type of plants (rigid or flexible). In their study, they found that the drag for flexible vegetation and for this submergence ratio was *C*_*D*_ = 0.39 compared to rigid plants, with *C*_*D*_ = 1. Considering this *C*_*D*_ for flexible plants, the threshold of ${\left[{C}_{D-Patch}\frac{n{d}^{2}}{2(1-\varnothing )}\right]}^\frac{2}{3}{{U}_{w}}^{2}$=2 would increase to ${\left[{C}_{D-Patch}\frac{n{d}^{2}}{2(1-\varnothing )}\right]}^\frac{2}{3}{{U}_{w}}^{2}$=3.8, being closer, therefore, to that obtained for rigid plants ${\left[{C}_{D-Patch}\frac{n{d}^{2}}{2(1-\varnothing )}\right]}^\frac{2}{3}{{U}_{w}}^{2}=4$.

As Pujol et al.^30^ pointed out, *TKE* production the correct diffusion of oxygen at the leaves’ boundary layer. The current study demonstrates that the behaviour the seagrass not only depends on the hydrodynamics, but also on the structural characteristics of the canopy, i.e., canopy density, patch length, and plant stiffness. Below the threshold of ${\left[{C}_{D-Patch}\frac{n{d}^{2}}{2(1-\varnothing )}\right]}^\frac{2}{3}{{U}_{w}}^{2}$, the behaviour of the canopy changes and its role is to reduce the seabed generated *TKE*. The current study also demonstrates that on the vertical axis, two regions can be differentiated for the flexible vegetation in terms of *TKE* behaviour. For the flexible vegetation and for ${\left[{C}_{D-Patch}\frac{n{d}^{2}}{2(1-\varnothing )}\right]}^\frac{2}{3}{{U}_{w}}^{2}>4$, the *TKE* in the UCL remains constant and is close to that for the non-vegetated cases. In this case, in the UCL the plants behave like blades, moving with the flow but not producing any additional *TKE* than that already present for the non-vegetated set-ups. In contrast, in the LCL, the *TKE* increases with ${\left[{C}_{D-Patch}\frac{n{d}^{2}}{2(1-\varnothing )}\right]}^\frac{2}{3}{{U}_{w}}^{2}$. In this case, plants in the LCL behave like stems, with small swaying movements, thus creating drag in the flow and producing *TKE*.

This result also aligns with that found by Bouma et al.^23^ when comparing the dissipation of wave height by *Spartina alternifora* to that of *Zostera noltii*. In their case, greater wave height dissipation was obtained for the more rigid *Spartina alternifora* vegetation. This is in accordance with Zhang et al.^20^ who divided the vertical structure of a flexible plant into two parts. The upper part was named the blade-like region and the lower part the stem-like region. In the stem-like region, they found a greater production of *TKE* compared with the blade-like region due to the greater relative motion between the flow velocity and the plant.

Contrary to flexible stems, rigid plants for ${\left[{C}_{D-Patch}\frac{n{d}^{2}}{2(1-\varnothing )}\right]}^\frac{2}{3}{{U}_{w}}^{2}>2$ present stem-like behaviour along the entire stem. In this case, *TKE* production is due to the greater relative motion between the flow and the rigid stem compared to the flexible blades. Contrary to flexible stems, in the UCL of rigid stems, *TKE* increases with ${\left[{C}_{D-Patch}\frac{n{d}^{2}}{2(1-\varnothing )}\right]}^\frac{2}{3}{{U}_{w}}^{2}$.From the results of the vertical attenuation of the *TKE* and the *TKE* attenuation compared to the non-vegetated cases, rigid plants exhibit a similar behaviour to flexible plants for high wave frequencies (*f* = 1.12 Hz). In contrast, under low wave frequencies, when flexible plants have a large sway movement, the hydrodynamics are far from those obtained by rigid plants.

Considering the thresholds for both rigid and flexible vegetation, the required canopy density to begin to produce TKE could be determined in terms of either the length of the patch or the canopy density. The ratio *TKE/Uw*^*2*^ was considered to range from 0.004 to 0.04 as was found in the laboratory tests. Four ratios *L*_*patch*_*/L*_*canopy*_ (see the “Methodology” section for the definition of *L*_*patch*_ and *L*_*canopy*_) will be considered, from 0.01 to 0.08. Considering these range of variation, flexible plants would require a canopy density ranging from 136 shoots m^−2^ to 6140 shoots m^−2^ (Fig. 5a). In contrast, a patch of rigid plants would require a density ranging from 69 shoots m^−2^ to 4046 shoots m^−2^ (Fig. 5b). Therefore, a patch of rigid plants would be capable of producing *TKE* in sparser canopy densities than a patch of flexible plants. This result might also have important implications for the sediment bed characteristics, with more provability of resuspension and scouring in regions covered with rigid canopies than in regions with flexible canopies when subject to high energetic conditions. This would align with the results of Bouma et al.^23^ who found that for hydrodynamic exposed areas, the flexible shoots of *Zostera* caused far less scouring than the stiff shoots of *Puccinellia*. In addition, a small patch of flexible plants would require a denser vegetation to produce the same normalized *TKE/U*_*w*_^*2*^ than a larger patch but with sparser vegetation. Therefore, the parameter ${\left[{C}_{D-Patch}\frac{n{d}^{2}}{2(1-\varnothing )}\right]}^\frac{2}{3}$ is related to the total effect of the vegetation patch in terms of drag, length and density.

## Conclusions

The current study presents the role plant flexibility plays, together with canopy density and patch length, in determining the hydrodynamics within a seagrass meadow. Flexible plants move with the flow in the upper part of the canopy layer but present a more rigid structure in the inner canopy layer. In contrast, canopies of rigid plants produce a high drag on the flow along the entire length of their stem, resulting in a turbulent kinetic energy production. This difference between the two canopy structures can explain their distribution in the environment, with rigid canopies being more extended in more sheltered regions, and flexible plants being more characteristic of more exposed regions with high flow energy. Rigid and flexible vegetation presents a similar stem-like behaviour in the inner part of the canopy for ${\left[{C}_{D-Patch}\frac{n{d}^{2}}{2(1-\varnothing )}\right]}^\frac{2}{3}{{U}_{w}}^{2}>2$ and ${\left[{C}_{D-Patch}\frac{n{d}^{2}}{2(1-\varnothing )}\right]}^\frac{2}{3}{{U}_{w}}^{2}>4,$ respectively, whereas in the canopy top layer flexible plants move with the flow to cope with the hydrodynamics, presenting a blade-like behaviour. In contrast, neither rigid nor flexible plants for ${\left[{C}_{D-Patch}\frac{n{d}^{2}}{2(1-\varnothing )}\right]}^\frac{2}{3}{{U}_{w}}^{2}<2$ or ${\left[{C}_{D-Patch}\frac{n{d}^{2}}{2(1-\varnothing )}\right]}^\frac{2}{3}{{U}_{w}}^{2}<4,$ respectively, produce turbulent kinetic energy. In addition, the behaviour of flexible plants might also move to being closer to that of rigid plants for high wave frequencies. In contrast, flexible plants produce a larger sway movement when they are under low oscillatory frequencies.

All in all, seagrass canopies are ecological engineers that modify the physical environment or, conversely, their distribution and extension depend on the trade-off between their physiological demands and their ability to withstand the energy of the system.

## Methodology

### The flume

**Figure 6:**
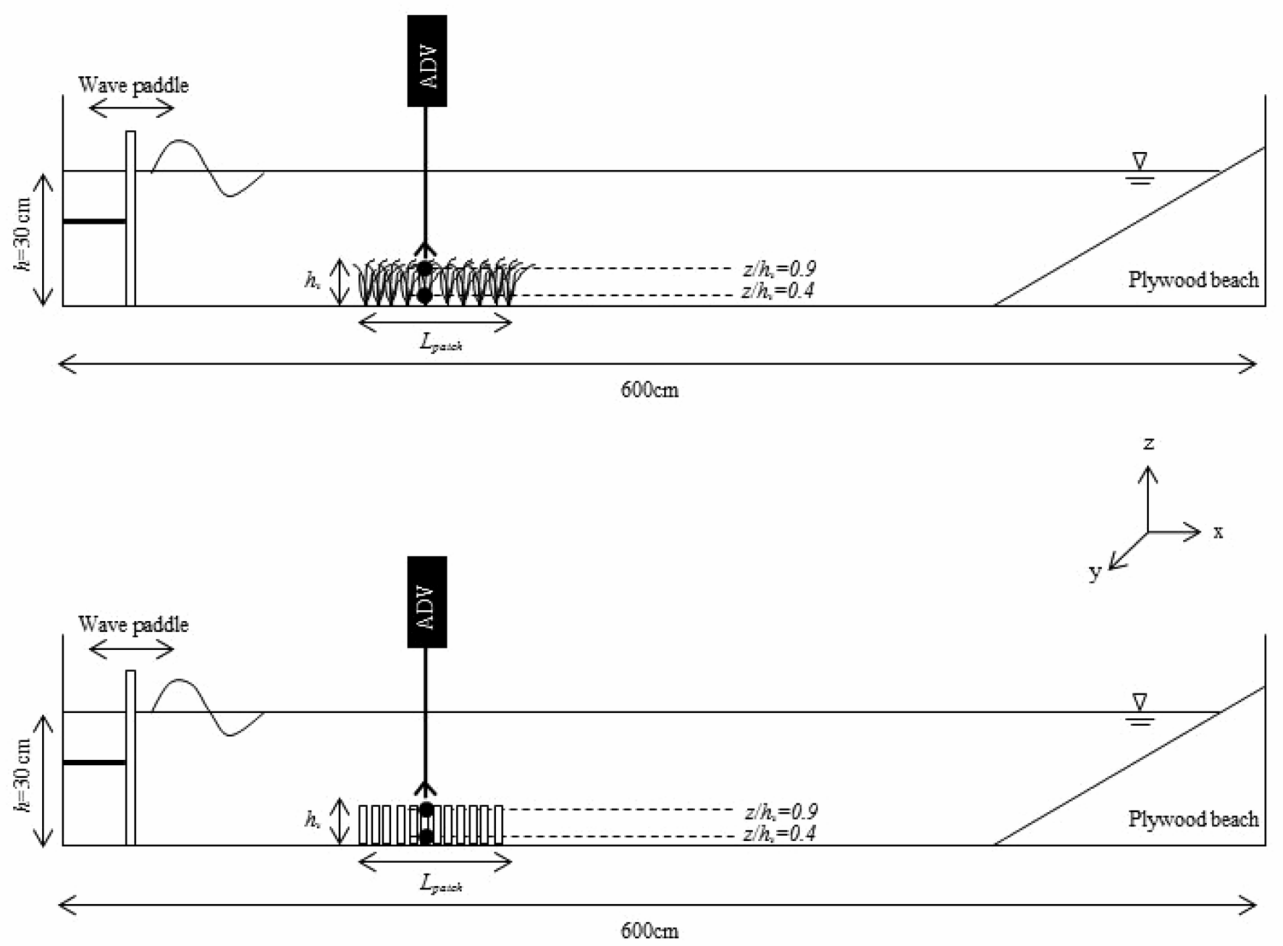
Lateral view of the experimental setup, with the wave paddle on the left. Experiments were conducted in a 600 × 50 × 50 cm long flume, with a mean water depth of 30 cm. The model patch had lengths that ranged from 2.8 to 42 cm and a patch height of effective height *h*_*v*_. The triangle at the water–air interface represents the water level in the flume. An ADV was vertically mounted to measure the instantaneous velocities at selected vertical heights. The upper panel corresponds to the case of flexible plants and the bottom panel to rigid plants.

The study was carried out in a laboratory methacrylate flume (600 cm long, 50 cm wide, and 60 cm deep, Fig. 6) with a mean water height of *h* = 30 cm (Table 1). The flume was equipped with a vertical paddle-type wavemaker at the entrance. The wavemaker was driven by a variable-speed motor at two frequencies (*f* = 0.5 Hz, 1.12 Hz). Wave heights measured by a wave gauge indicated that wave amplitudes were 6 cm and 3 cm for wave frequencies of 1.12 Hz and 0.5 Hz, respectively. A plywood beach (slope = 1:2) was placed at the end of the flume and covered with foam rubber to eliminate wave reflection^2,30^. In the longitudinal direction, *x* = 0 cm was situated at the wavemaker, in the lateral direction, *y* = 0 cm was in the centre of the tank, and in the vertical direction, *z* = 0 cm was situated at the flume bed.

**Table 1:** Nomenclature table.

Variable	Units	Definition	Variable	Units	Definition
*A*_*w*_	cm	Wave excursion length	*u'*	cm s^−1^	Turbulent velocity
*A*_*w*_*/S*	Non-dimensional	Ratio between wave excursion to plant-to-plant distance	*U*_*c*_	cm s^−1^	Steady velocity associated with the current
*C*_*D*_	Non-dimensional	Drag of the obstacle along the fluid	*U*_*i*_	cm s^−1^	Instantaneous velocity
*d*	cm	Stem diameter	*U*_*i*_*(φ)*	cm s^−1^	Instantaneous velocity according to the phase
*f*	Hz	Wave frequency	*U*_*w*_	cm s^−1^	Wave velocity
*h*	cm	Water height	*U*_*w*_^*rms*^	cm s^−1^	Orbital velocity
*h*_*v*_	cm	Canopy height	*v*	cm s^−1^	Eulerian velocity in the *y* direction
*L*_*canopy*_	cm	Canopy length	*x*	cm	Longitudinal direction
*L*_*patch*_	cm	Patch length	*x* = *0*	cm	Position of the wave paddle
*n*	stems m^−2^	Canopy density	*y*	cm	Lateral direction
*S*	cm	Plant-to-plant distance	*z*	cm	Vertical direction
*SPF*	%	Solid plant fraction	*β'*	Non-dimensional	Vertical ratio between the *TKE* at the canopy top layer and the inner canopy layer
*TKE*	cm^2^ s^−2^	Turbulent kinetic energy	*β*_*UCL*_ and *β*_*LCL*_	Non-dimensional	Ratio between the *TKE* with vegetation and without vegetation for both, the upper canopy layer, and the lower canopy layer
*u*	cm s^−1^	Eulerian velocity in the *x* direction	*ϕ*	Non-dimensional	Solid volume fraction
LCL	–	Lower-canopy layer	UCL	–	Upper-canopy layer
ACL		Above-canopy layer	WP	–	Non-vegetation set up
*h*_*p*_	cm	Leaf length for flexible vegetation and stem length for rigid vegetation			

### Patches of flexible vegetation

Two types of submerged vegetation models, rigid and flexible, were used (Fig. 6). The rigid vegetation (SRV) consisted of a series of 1 cm thick 14 cm high PVC dowels. The flexible vegetation (SFV) consisted of a series of flexible plants of eight 0.075 mm thick polyethylene canopy blades attached to PVC dowels 1 cm in diameter and 2 cm high that had been randomly inserted into a perforated baseboard^2^ (250 cm in length), with the rigid dowel extending 1 cm above the bed^20^. The plants in the flexible model were geometrically and dynamically similar to *Posidonia oceanica* plants^2,31^. The plant leaves in the flexible vegetation model were of 14 cm long. However, the effective height for the flexible vegetation when the leaves were bent by the waves was *h*_*v*_ = 8.5 cm for the wave frequency *f* = 1.12 Hz and *h*_*v*_ = 10.5 cm for the wave frequency *f* = 0.5 Hz. In contrast, the effective height for the rigid plants was the length of the PVC dowel, *h*_*p*_ = 14 cm. The effective heights were calculated as the mean between both the maximum and minimum bending heights of the plants for 25 oscillations. From the observations, the effective plant height increased as the wave frequency decreased. A linear fit between these two data points was made (*h*_*v*_ = − 3.23f + 12.11). For the other studies considered here, the effective plant height was not always available, but it was estimated by the linear fit above between *h*_*v*_ and *f*.

The density of the vegetated patches was quantified using the solid plant fraction (*SPF*) defined as:

1 $$SPF (\%)=100n \pi {\left(\frac{{d}}{{2}}\right)}^{2}$$

where *n* is the number of stems per unit area and *d* is the stem diameter (1 cm). Therefore, *SPF* represents the percentage of vegetation covering the base to the flume. For the rigid vegetation three *SPFs* were used (0%, 3.5% and 10%) and for the flexible vegetation six *SPF*s were used (0%, 2.5%, 3.5%, 5%, 7.5% and 10%). These *SPF*s corresponded to vegetation densities of *n* = 0, 318, 446, 637, 955 and 1273 stems m^−2^ that are in the range of canopy densities found in coastal areas (78–1000 stems m^−2^)^6,12,32,33^. *SPF* = 0% corresponded to the case with no vegetation. For each *SPF* different patch sizes, *L*_*patch*_, were considered, with *L*_*patch*_ ranging from 42 to 245 cm, corresponding to 2 to 17 time the leaf length (Table 2). To determine *L*_*patch*_ in the experiments, the patch edge was considered as the interface between the vegetated and the non-vegetated regions. Thus, for the different *SPF*s, *L*_*patch*_, and the two wave frequencies, a total of 87 experiments were performed (Table 2).

**Table 2:** Summary of the experimental conditions tested.

Run	*f* (Hz)	*SPF* (%)	*n* (stems m^−2^)	*L*_*patch*_ (cm)	*A*_*w*_*/S* LCL	*A*_*w*_*/S* UTL	Run	*f* (Hz)	*SPF* (%)	*n* (stems m^−2^)	*L*_*patch*_ (cm)	*A*_*w*_*/S* LCL	*A*_*w*_*/S* UCL	Run	*f* (Hz)	*SPF* (%)	*n* (stems m^−2^)	*L*_*patch*_ (cm)	*A*_*w*_*/S* LCL	*A*_*w*_*/S* UCL
WP1	0.5	0	0				SFV35	1.12	3.5	446	112	0.24	0.28	SRV69	0.5			70	0.51	0.55
WP2	1.12	0	0				SFV36				126	0.24	0.28	SRV70				126	0.57	0.57
SFV3	0.5	1	127	42	0.33	0.35	SFV37				140	0.24	0.28	SRV71				182	0.52	0.55
SFV4				70	0.33	0.35	SFV38				154	0.24	0.28	SRV72				238	0.49	0.50
SFV5				112	0.33	0.34	SFV39				168	0.24	0.29	SRV73		10	1237	42	0.92	1.02
SFV6				196	0.33	0.33	SFV40				196	0.24	0.28	SRV74				70	0.95	1.04
SFV7		7.5	955	42	1.01		SFV41				238	0.24	0.28	SRV75				126	0.92	1.03
SFV8				70	0.97	0.99	SFV42		5	637	42	0.29	0.36	SRV76				182	0.91	1.01
SFV9				112	0.98	0.96	SFV43				70	0.29	0.37	SRV77				238	0.85	0.96
SFV10				196	0.94	0.94	SFV44				98	0.29	0.36	SRV78	1.12	3.5	446	42	0.23	0.30
SFV11		10	1273	42	0.83	1.11	SFV45				126	0.29	0.36	SRV79				70	0.23	0.30
SFV12				70	0.83	1.06	SFV46				168	0.28	0.36	SRV80				126	0.27	0.32
SFV13				84	0.79	1.13	SFV47				196	0.28	0.35	SRV81				182	0.23	0.29
SFV14				98	0.79	1.09	SFV48				210	0.28		SRV82				238	0.26	0.32
SFV15				112	0.78	1.25	SFV49				238	0.28	0.35	SRV83		10	1273	42	0.32	0.38
SFV16				133	0.80	1.14	SFV50		7.5	955	42	0.36	0.44	SRV84				70	0.43	0.50
SFV17				140	0.76	1.13	SFV51				70	0.37	0.45	SRV85				126	0.38	0.47
SFV18				154	0.80	1.10	SFV52				84	0.36	0.45	SRV86				182	0.39	0.45
SFV19				182	0.81	1.13	SFV53				98	0.36	0.44	SRV87				238	0.35	0.40
SFV20				224	0.77	1.09	SFV54				112	0.35	0.44							
SFV21				238	0.77	1.08	SFV55				126	0.35	0.45							
SFV22	1.12	2.5	318	42	0.21	0.25	SFV56				154	0.35	0.44							
SFV23				70	0.21	0.25	SFV57				196	0.34	0.44							
SFV24				84	0.21	0.25	SFV58				238	0.34	0.42							
SFV25				98	0.21	0.25	SFV59		10	1273	42	0.41	0.49							
SFV26				112	0.21	0.24	SFV60				70	0.41	0.52							
SFV27				126	0.21	0.24	SFV61				84	0.40	0.52							
SFV28				140	0.21	0.24	SFV62				98	0.39	0.52							
SFV29				154	0.21	0.24	SFV63				126	0.38	0.50							
SFV30				168	0.21	0.24	SFV64				154	0.39	0.50							
SFV31				182	0.21	0.24	SFV65				168	0.39	0.52							
SFV32				196	0.21	0.24	SFV66				196	0.40	0.51							
SFV33				238	0.21	0.24	SFV67				238	0.37	0.47							
SFV34	1.12	3.5	446	70	0.25	0.29	SRV68	0.5	3.5	446	42	0.53	0.54							

Where SFV correspond to Submerged Flexible Vegetation and SRV to Submerged Rigid Vegetation. LCL denotes the lower canopy layer and UCL the upper canopy layer.

### Measuring velocities

The Eulerian velocity field was defined as (*u*, *v*, *w*) in the (*x*, *y*, *z*) directions, respectively. The three components of velocity were recorded for 5 min at a measuring frequency of 50 Hz with a downwards looking Acoustic Doppler Velocimeter (16-MHz MicroADV, Sontek). The ADV was mounted on a movable vertical frame (at *y* = 0 cm, Fig. 1) and manually adjusted to measure a vertical profile. Some plants were removed (and re-inserted into nearby holes) to avoid blocking the ADV beams^20,34,35^. The ADV measured at a 5 cm distance from the probe tip, and with a sampling volume of 0.09 cm^3^. The longitudinal velocity was measured at an antinode to eliminate the lower order spatially periodic variation in wave and velocity amplitude associated with wave reflection^2,36^. Beam correlations less than 70% were discarded and spikes were removed^2,37^.

### Hydrodynamic analysis

For oscillatory flows, the instantaneous velocity in the *x* direction, *U*_*i*_*(t)*, can be decomposed as:

2 $${{U}}_{{i}}{(t)} ={{U}}_{{c}}+ {{U}}_{{w}}+ {u^{\prime}}$$

where *U*_*c*_ is the steady velocity associated with the wave, *U*_*w*_ is the unsteady wave motion in the *x* direction which represents spatial variations in the phase-averaged velocity field, and *u′* is the turbulent velocity, that is, the instantaneous velocity fluctuation in the x-direction. *U*_*c*_ is the phase-averaged velocity:

3 $${{U}}_{{c}}= \frac{1}{{2\pi}}{\int }_{0}^{2\pi}{{U}}_{{i}}\left( \varphi \right) \partial \varphi$$

where ${{U}}_{{i}}\left(\varphi\right)$ is the instantaneous velocity according to the phase^36^. Wave velocity, *U*_*w*_, was obtained by using a phase averaging technique. The Hilbert transform was used to average oscillatory flow velocities with a common phase^16,35^. The root mean square (rms) of ${{U}}_{{i}}\left(\varphi\right)$ was considered as the characteristic value of the orbital velocity *U*_*w*_^*rms*^ (*U*_*w*_ hereafter) at each depth, and was calculated according to:

4 $${{U}}_{{w}}^{rms} = \sqrt{\frac{1}{{2} \pi }{\int }_{0}^{{2} \pi }{{(}{{U}}_{{i}}\left(\varphi\right)-{{U}}_{{c}}{)}}^{2} \partial\varphi}$$

The turbulent velocity was obtained by:

5 $${{u}}^{{\prime}}= {{ U}}_{{i}} - {{U}}_{{c }}-{{U}}_{{w}}$$

where *U*_*c*_ and *U*_*w*_ were calculated by Eqs. (3) and (4). The turbulent velocity was calculated for all directions (*u′*, *v′,* and *w′*) for z = 4 cm.

Following Ros et al.^35^, turbulent kinetic energy (*TKE*) was calculated as:

6 $$TKE = \frac{1}{{2}}\left(\langle {{{u}}^{{\prime}}}^{2}\rangle + \langle {{{v}}^{{\prime}}}^{2}\rangle + \langle {{{w}}^{{\prime}}}^{2}\rangle \right)$$

where <  > denotes the average over the wave phase.

The ratio, *β*, was calculated following Colomer et al.^6^ for both, the UCL and the LCL:

7 $${\beta}_{UCL} = \frac{{TKE_{{UCL}} }}{{TKE_{{WP,UCL}} }} \; \; \text{and} \;\; {\beta}_{LCL} = \frac{{TKE_{{LCL}} }}{{TKE_{{WP,LCL}} }}$$

where ${{TKE}}_{UCL}$ ${{TKE}}_{LCL}$ were the turbulent kinetic energy values in the UCL and LCL, respectively. For the ${{TKE}}_{UCL}$, *TKE* at *z* = 12 cm was the characteristic TKE considered, whereas for the ${{TKE}}_{LCL}$, TKE at z = 4 cm was considered. For the non-vegetated cases and ${{TKE}}_{{WP}}$,the *TKE* considered was also that measured at *z* = 12 cm and z = 4 cm, respectively. Therefore, the values of ${\beta}_{UCL} \approx 1 \;\; {\text{and}} \;\; {\beta}_{UCL} \approx 1$ indicated a weak or negligible attenuation of the *TKE*, whereas low values of ${\beta}_{UCL} <1 \; \; {\text{and}} \; \; {\beta}_{UCL} < {1 }$ indicated a high *TKE* attenuation compared to the non-vegetated case.

The vertical *TKE* attenuation was calculated as *β′*:

8 $${\beta }^{{\prime}} = \frac{{{TKE}}_{LCL}}{{{TKE}}_{LCL}}$$

where ${{TKE}}_{LCL}$ and ${{TKE}}_{{UCL}}$ were the turbulent kinetic energies in the LCL and UCL, respectively. For ${{TKE}}_{{UCL}}$ , the *TKE* at *z* = 4 cm was considered the characteristic *TKE* of this layer, whereas the *TKE* measured at *z* = 12 cm was the characteristic *TKE* for the UCL. Therefore, values of ${\beta }^{{\prime}}{ \approx 1}$ indicated a weak or negligible vertical attenuation of the *TKE*, whereas low values of ${\beta }^{{\prime}}{ <1}$ indicated a high *TKE* vertical attenuation, meaning greater *TKE* at *z* = 4 cm comparted to *z* = 12 cm.

To gain knowledge about the vertical distribution of *TKE* within the patch, a model was set up following Zhang et al.^20^. For a full canopy, Zhang et al.^20^ found that the relationship between the *TKE*, *U*_*w,*_ and the main canopy parameters followed:

9 $$\frac{TKE}{{{U}_{w}}^{2}}=\delta {\left[{C}_{D}\frac{{l}_{t}}{d}\frac{n{d}^{2}}{2\left(1-\upphi \right)}\right]}^\frac{2}{3}$$

where δ is the scale constant, ϕ is the solid volume fraction, $\phi =n\frac{\pi }{4}{d}^{2}$, *l*_*t*_ is characteristic eddy length-scale, and *C*_*D*_ is the drag of the form of the obstacle along with the fluid patch, with *C*_*D*_ = 1.4 being considered in the study. In Eq. (9), the characteristic length scale, *L*_*patch*_/*L*_*canopy*_ is introduced to account for the volume of the patch in relation to the maximum canopy volume in the form of ${\left(\frac{{L}_{patch}}{{L}_{canopy}}\right)}^\frac{1}{3}$. *L*_*canopy*_ was considered as the length of the vegetation patch from where the wave velocity did not change with a further increase in its length. Barcelona et al.^29^ found that *L*_*canopy*_ depended on the wave frequency, *f*, with *L*_*canopy*_ = 20*h*_*v*_ for *f* = 1.12 Hz and *L*_*canopy*_ = 10*h*_*v*_ for *f* = 0.5 Hz. Therefore Eq. (9) is expressed following:

10 $$\frac{TKE}{{{U}_{w}}^{2}}=\delta {\left[{C}_{D}{\left(\frac{{L}_{patch}}{{L}_{canopy}}\right)}^\frac{1}{3}\frac{{l}_{t}}{d}\frac{n{d}^{2}}{2\left(1-\upphi \right)}\right]}^\frac{2}{3}$$

Zhang et al.^20^ considered *l*_*t*_ = *d* for *S* > *2d* whereas *l*_*t*_ = *S* for *S* < *2d*. In the present study, *S* > *2d*, *l*_*t*_ = *d*. Therefore,

11 $$\frac{TKE}{{{U}_{w}}^{2}}=\delta {\left[{C}_{D}{\left(\frac{{L}_{patch}}{{L}_{canopy}}\right)}^\frac{1}{3}\frac{n{d}^{2}}{2\left(1-\upphi \right)}\right]}^\frac{2}{3}$$

The parameter *ϕ* has been substituted by its definition to obtain two differentiated parameters (one related to patch length and the other to shoot density), as:

12 $$\frac{TKE}{{{U}_{w}}^{2}}=\delta {\left[{\left(\frac{{L}_{patch}}{{L}_{canopy}}\right)}^\frac{1}{3}\frac{2{C}_{D}n{d}^{2}}{4-\pi n{d}^{2}}\right]}^\frac{2}{3}$$

To obtain a more complete model the experiments from Zhang et al.^20^, Barcelona et al.^29^ and Pujol et al.^16^ were added to the comparison (Table 3).

**Table 3:** Summary of the experimental conditions tested by Zhang et al.^20^, Barcelona et al.^29^ and Pujol et al.^16^.

	Run	*f* (Hz)	*SPF* (%)	*n* (stems·m^−2^)	*L*_*patch*_ (cm)	*Aw/S UC*L		Run	*f* (Hz)	*SPF* (%)	*n* (stems·m^−2^)	*L*_*patch*_ (cm)	*Aw/S UC*L		Run	*f* (Hz)	*SPF* (%)	*n* (stems·m^−2^)	*L*_*patch*_ (cm)	*Aw/S UC*L	*Aw/S LC*L
Barcelona et al.^37^	B. SFV 1	0.7	1	127	245	0.03	Zhang et al.^20^	Z. SFV 1	1	1.1	280	200	1.21	Pujol et al.^16^	P.SRV 1	0.8	1	127	245	0.07	0.06
	B. SFV 2				245	0.04		Z. SFV 2				200	0.94		P.SRV 2		5	637	245	0.18	0.15
	B. SFV 3				245	0.11		Z. SFV 3				200	0.74		P.SRV 3		10	1280	245	0.25	0.18
	B. SFV 4				245	0.09		Z. SFV 4				200	0.54		P.SRV 4	1	1	127	245	0.05	0.07
	B. SFV 5		2.5	318	245	0.07		Z. SFV 5				200	0.42		P.SRV 5		5	637	245	0.13	0.14
	B. SFV 6				245	0.03		Z. SFV 6				200	0.29		P.SRV 6		10	1280	245	0.16	0.22
	B. SFV 7				245	0.15		Z. SFV 7		2.3	600	200	2.01		P.SRV 7	1.4	1	127	245	0.04	0.06
	B. SFV 8				245	0.17		Z. SFV 8				200	1.52		P.SRV 8		5	637	245	0.08	0.14
	B. SFV 9		5	637	245	0.04		Z. SFV 9				200	1.21		P.SRV 9		10	1280	245	0.11	0.17
	B. SFV 10				245	0.07		Z. SFV 10				200	0.88		P.SFV 1	0.8	1	127	245	0.06	0.06
	B. SFV 11				245	0.12		Z. SFV 11				200	0.68		P.SFV 2		5	637	245	0.14	0.15
	B. SFV 12				245	0.12		Z. SFV 12				200	0.46		P.SFV 3		10	1280	245	0.15	0.19
	B. SFV 13		7.5	955	245	0.11		Z. SFV 13		3.2	820	200	2.01		P.SFV 4	1	1	127	245	0.06	0.08
	B. SFV 14				245	0.06		Z. SFV 14				200	1.66		P.SFV 5		5	637	245	0.12	0.17
	B. SFV 15				245	0.07		Z. SFV 15				200	1.40		P.SFV 6		10	1280	245	0.26	0.21
	B. SFV 16				245	0.14		Z. SFV 16				200	1.06		P.SFV 7	1.4	1	127	245	0.04	0.06
	B. SFV 17	1.2	1	127	245	0.07		Z. SFV 17				200	0.80		P.SFV 8		5	637	245	0.10	0.14
	B. SFV 18				245	0.04		Z. SFV 18				200	0.49		P.SFV 9		10	1280	245	0.13	0.19
	B. SFV 19				245	0.05		Z. SFV 19		5.3	1370	200	2.22								
	B. SFV 20				245	0.04		Z. SFV 20				200	1.90								
	B. SFV 21		2.5	318	245	0.06		Z. SFV 21				200	1.57								
	B. SFV 22				245	0.07		Z. SFV 22				200	1.19								
	B. SFV 23				245	0.08		Z. SFV 23				200	0.91								
	B. SFV 24				245	0.08		Z. SFV 24				200	0.55								
	B. SFV 25		5	637	245	0.08															
	B. SFV 26				245	0.10															
	B. SFV 27				245	0.15															
	B. SFV 28				245	0.12															
	B. SFV 29		7.5	955	245	0.13															
	B. SFV 30				245	0.14															
	B. SFV 31				245	0.15															
	B. SFV 32				245	0.15															

## Data Availability

Data will be accessible from the following public data repository link: 10.34810/data528.
